# Parenting Behavior at 18 Months Predicts Internalizing and Externalizing Problems at 6 Years in Moderately Preterm and Full Term Children

**DOI:** 10.3390/ijerph17228679

**Published:** 2020-11-23

**Authors:** Lisa Oosterom, Lilly Bogičević, Marjolein Verhoeven, Anneloes L. van Baar

**Affiliations:** Child and Adolescent Studies, Utrecht University, Heidelberglaan 1, 3584 CS Utrecht, The Netherlands; l.a.oosterom@uu.nl (L.O.); l.bogicevic@uu.nl (L.B.); j.c.t.verhoeven@uu.nl (M.V.)

**Keywords:** behavior problems, moderately preterm, prematurity, parenting behavior, mother–child interaction

## Abstract

Moderately preterm born children (MPT) are at increased risk for behavior problems compared to full term born (FT) children. MPT children may receive less optimal parenting, and in response, may develop behavior problems. Our aims were to examine whether parenting behavior and mother–child interaction quality mediate the association between birth status and child behavior problems. Participants were 120 MPT children and 100 FT children. At 18 months of age, mothers reported on their parenting behavior (support and structure), and mother–child interaction (sensitivity and limit-setting) was observed. At 6 years of age, mothers reported on children’s behavior problems. Using structural equation modeling, birth status was found to predict attention problems, but not internalizing and externalizing problems. Mothers of MPT children set less appropriate limits than mothers of FT children at 18 months of age. More maternal structure at 18 months predicted fewer internalizing and externalizing problems, but not attention problems, at 6 years. These associations between parenting behavior, mother–child interaction quality, and child behavior problems were similar for MPT and FT children. Our findings indicate that maternal structure in toddlerhood is an important predictor of later internalizing and externalizing problems for both MPT and FT children.

## 1. Introduction

Worldwide, 10.6% of all children are born preterm [1]. Preterm births are births that occur before 37 weeks of gestation [2], and are often further classified into three subcategories: extremely preterm (<28 weeks’ gestational age (GA)), very preterm (28–32 weeks’ GA), and moderately preterm (32–37 weeks’ GA) [2]. Even though moderate preterm births account for 84.7% of all preterm births [1], most research has focused on the development of very and extremely preterm born children. Although moderately preterm born (MPT) children experience fewer neonatal complications than very preterm born children, recent studies show they are still at elevated risk for breathing difficulties, hypoglycemia, and feeding problems compared to full term born (FT) children [3]. Additionally, at school age, MPT children show more cognitive, school, and behavior problems than their full term born peers [4].

Previous research suggests that MPT children experience elevated levels of behavior problems, although results differ in the severity and nature of these problems. To illustrate, in one study, MPT children had higher scores than FT children on all syndrome scales, and the internalizing, externalizing, and total problems scales of the Child Behavior Checklist [5], whereas other studies found MPT children to only show higher levels of attention problems and internalizing problems [6,7], or found no differences at all [8]. Behavior problems can negatively affect academic and psychological functioning [9], and they often persist into adolescence and adulthood [10]. Therefore, to better understand the etiology of behavior problems in MPT children, it is important to identify factors that affect these behavior problems. To date, long-term follow-up studies examining predictors of behavior problems in MPT children are rare, and relatively little is known about early developmental precursors of these behavior problems.

One explanation for the elevated risk for behavior problems in MPT children is preterm birth itself. Preterm birth occurs at a time of rapid brain development and increases the risk for early acquired brain injury and disruption to normal brain development [11]. At birth, the brains of MPT infants are smaller and less mature than the brains of FT infants [12]. The relative immaturity of MPT infants and neonatal complications such as respiratory difficulties, cardiac complications, and infections also increase the risk for brain injury [11]. Regional disruptions in brain development detectable shortly after birth in preterm children are, in turn, associated with impaired social–emotional development at school age, as well as with cognitive, neurosensory, and language impairment [13,14,15]. However, according to Sameroff’s Unified Theory of Development [16], development is not only an outcome of biological processes, but is also influenced by children’s interactions with their (social) environment.

The parent–child relationship forms one of the most important social environments in which child development occurs and parenting behavior and parent–child interaction quality may explain elevated levels of behavior problems in MPT children. Parental control and parental warmth represent two broad dimensions of parenting that may play a role in the development of child behavior problems [17]. Parental control includes aspects of parenting behavior such as monitoring children’s behavior, using consistent discipline, providing structure and guidelines for behavior, and limit-setting. Parental warmth is characterized by positive affect, responsivity, support, and sensitivity to children’s needs [18]. Parental limit-setting and behavioral control are related to lower levels of externalizing and internalizing problems [19]. However, negative parental control and discipline, such as anger, harshness, criticism, and excessive or intrusive control, increase the risk for the child’s development of behavior problems [19]. With regard to parental warmth, mothers’ sensitive behaviors were found to decrease the risk for internalizing and externalizing problems in their children [20,21]. A study by Laucht, Esser, and Schmidt [22] found that children born with low birth weight experienced more attention problems than children born with normal birth weight when mothers were emotionally unresponsive.

When studying the associations between parenting and child development, it is important to acknowledge that development is an outcome of dynamic interactions between children and their environment [16]. The child is not passively influenced by the environment, but also shapes this environment. Characteristics of preterm born children may elicit less optimal parenting behaviors, as these children tend to be less responsive, less attentive, more passive, and smile less frequently during mother–child interaction than full term born children [23,24,25]. Indeed, there is evidence that mothers of preterm born children are less sensitive and more controlling than mothers of FT children [23,24,26]. These parenting behaviors may further affect the child’s development, particularly during toddlerhood. Thus, less optimal parenting behaviors and a poorer parent–child interaction quality might develop in response to characteristics of the preterm child, which in turn might explain why MPT children are at increased risk for behavior problems. In the current study, we therefore examine if parenting behavior and mother–child interaction quality mediate the association between preterm birth and child behavior problems.

Parenting is important for all children, but certain characteristics, such as prematurity, could make children more susceptible to either the positive or negative influences of parenting [27]. For example, it was found that high levels of maternal emotional distress negatively affected the cognitive functioning of preterm infants, but not of FT infants [28]. In addition, compared to children with normal birth weight, children born with low birth weight were more susceptible to the adverse effects of low-sensitive parenting, but not to the beneficial effects of high-sensitive parenting on their academic achievement [29]. Some evidence suggests that preterm born children are not only more strongly affected by low quality parenting, but also by high quality parenting. This is in line with the susceptibility hypothesis [30] For example, preterm infants’ social competence was more strongly influenced by both high and low levels of maternal stress and low- and high-quality triadic interaction than that of FT infants [28]. The results of these studies suggest that children born at risk may be more strongly affected by their early caregiving environment than children born without additional risk. In contrast, some studies found that full term infants [31] and low medical risk infants [32] were more susceptible to the effects of parenting than preterm born and high medical risk infants. The moderating role of birth status in the associations between parenting and child behavior problems has not yet been investigated for MPT children specifically, and studies investigating this effect in other high-risk populations yielded inconsistent results.

**Figure 1:**
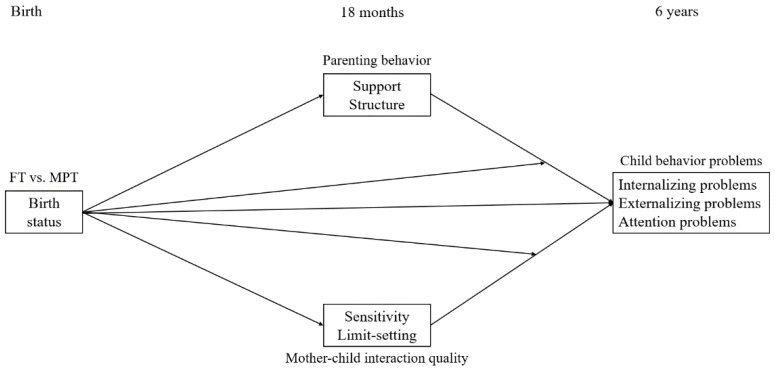
Conceptual model of the project. Birth status: 0 = full term, 1 = moderately preterm. FT = full term, MPT = moderately preterm.

The present study examines MPT and FT children’s behavior problems in relation to parenting at toddler age. It is based on data from the STAP project (Study on Attention of Preterm children). A previous study, using different data from this project, examined cognitive and behavioral functioning at 6 years of age, and reported higher levels of attention problems, but not internalizing and externalizing problems, in MPT children compared with FT children [33]. The present study includes data on parenting behavior and mother–child interaction at 18 months and employs structural equation modeling to examine the predictive value of birth status in relation to child behavior problems, aiming to explain these relations. The aim of the present study is threefold: (1) to assess whether birth status predicts child behavior problems at 6 years of age, (2) to determine whether parenting behavior and mother–child interaction quality mediate the association between birth status and child behavior problems, and (3) to examine whether birth status moderates the associations of parenting behavior and mother–child interaction quality with child behavior problems. First, we expect that MPT children experience more behavior problems than FT children at 6 years of corrected age. Our second expectation is that parenting behavior and mother–child interaction quality at 18 months of corrected age will mediate the association between birth status and child behavior problems. Lastly, we expect that birth status will moderate the associations of parenting behavior and mother–child interaction quality with child behavior problems. The model studied is presented in Figure 1.

## 2. Materials and Methods

### 2.1. Participants

Participants were children from the longitudinal STAP Project, born between March 2010 and April 2011. Children were recruited from nine hospitals around Utrecht, the Netherlands. Parents were invited to participate through their pediatrician or midwife when children were 10 months old. Exclusion criteria were admission to a tertiary Neonatal Intensive Care Unit (NICU), dysmaturity (birth weight below the 10th percentile according to Dutch reference curves [34]), multiple births, severe congenital malformations, maternal antenatal alcohol or drug abuse, and maternal chronic antenatal use of psychiatric drugs.

At the start of the project, 226 families agreed to participate. The present study included children who had data on at least one assessment at 18 months or 6 years (*N* = 220). At 18 months of age, 215 (95.1%) children had complete data for structure, and 214 (94.7%) children had complete data for support, sensitivity, and limit-setting. At 6 years of age, 157 (69.5%) children had complete data for child behavior problems. Of the 226 children who agreed to participate at the start of the project, 18 withdrew by 6 years of age, 5 were uncontactable, and 25 declined participation for this assessment wave. Children who did not have data at the age of 6 years did not differ from children who remained in the study in terms of birth status, gestational age, birth weight, gender, maternal education, sensitivity, limit-setting, support, and structure. In the present study, 220 (97.3%) children were included, of which 120 were MPT and 100 FT.

### 2.2. Procedure

Approval was obtained from the medical ethics committee of the University Medical Center Utrecht, identification code NL34143.041.10. Written informed consent was obtained from the mothers and fathers of all children. MPT children were invited for all assessments at corrected age to eliminate subtle maturational effects and optimize comparison with FT children. When children were 18 months of corrected age, mothers visited the lab at Utrecht University, where mother–child interaction was observed and videotaped. This mother–child interaction task lasted for 15 min. Afterwards, these videos were coded. If children would indicate they did not want to participate, the mother–child interaction task would be discontinued. During their visit at Utrecht University, mothers also filled out a paper-and-pencil questionnaire about their parenting behavior. At 6 years of corrected age, mothers reported on children’s behavior problems through an online questionnaire. All children received a small gift for participation and parents’ travel expenses were compensated.

### 2.3. Measures

#### 2.3.1. Parenting Behavior

At 18 months of corrected age, mothers completed the Comprehensive Early Childhood Parenting Questionnaire (CECPAQ) [35], a parent report measure of commonly occurring behaviors across five domains of parenting (i.e., support, stimulation, structure, harsh discipline, and positive discipline). The CECPAQ consists of 54 items describing parenting behaviors. Parents indicate the frequency of these behaviors on a 6-point Likert-scale, ranging from 1 (never) to 6 (always). Only the subscales support and structure were used in the present study. The subscale Support comprises 13 items, of which 4 items assess sensitivity (e.g., “I listen to my child’s feelings and understand them”), 5 items assess Responsiveness (e.g., “I am able to comfort my child when s/he is scared”), and 4 items assess Affection (e.g., “I hug, kiss, or hold my child for no particular reason”). The subscale structure consists of 12 items, assessing Consistency (3 items, e.g., “When my child misbehaves, I let my child out of punishment early”), Overreactivity, (4 items, e.g., “When my child misbehaves, I handle it without getting upset”), and Laxness (5 items, e.g., “When I say my child can’t do something, I let my child do it anyway”). Higher scores represent more optimal parenting behavior. Preliminary evidence for the psychometric quality of the CECPAQ was found [35. In the present study, internal consistency of the subscale support was good (α = 0.85) and internal consistency of the subscale structure was acceptable (α = 0.79).

#### 2.3.2. Mother–child Interaction Quality

Mother–child interaction was observed when children were 18 months of corrected age. Mothers were asked to play with their child for 15 min, which consisted of 5 min of free play and 10 min of structured play (i.e., reading a book and making a puzzle, each 5 min). The interaction was videotaped and coded afterwards. Coders were trained and blinded for gestational age of the children. Videos were coded with the Coding Interactive Behavior Manual (CIB) [36]. The CIB is a global rating system of parent–child interaction and includes 42 items assessing the frequency of specific behaviors: 21 for parents, 16 for infants, and 5 for dyads, rated on a 5-point scale ranging from 1 (rare) to 5 (often). In the present study, the parent items were combined into two CIB parent constructs. The first construct is maternal sensitivity, which includes the following items: acknowledgement of child’s interactive signals, positive affect, warm and clear vocal quality, appropriate range of affect, creativity/resourcefulness, supportive presence, and adaptation to the child’s needs and changing communications. The second construct is limit-setting, which consists of three items: consistency of style, on-task persistence, and appropriate structure/limit-setting. For both parent constructs, higher scores indicate higher levels of mother–child interaction quality. The CIB has been validated in several studies [37,38]. In the present study, internal consistencies of the sensitivity construct (α = 0.79) and the limit-setting construct (α = 0.71) were acceptable. Interrater reliability was acceptable with an intraclass correlation of 0.76 based on 21% double coded videos

#### 2.3.3. Behavior Problems

Mothers reported on their child’s behavior problems when children were 6 years of corrected age, using the Dutch version of the Child Behavior Checklist (CBCL/6–18) [39]. The CBCL/6–18 consists of two broadband scales (internalizing and externalizing behavior), and eight syndrome scales. In the present study, the broadband scales and the attention problems syndrome scale were used. The internalizing broadband scale includes anxious/depressed behavior, withdrawn/depressed behavior, and somatic complaints. The externalizing broadband scale includes rule-breaking behavior and aggressive behavior. Mothers indicated whether and how frequently their child displayed various behaviors for a total of 113 items on a 3-point Likert-scale, with 0 (not true), 1 (somewhat/sometimes), or 2 (very/often true). Raw scores were transformed to standardized T scores based on age and gender norms. Higher scores indicate more behavior problems. The broadband and syndrome scales of the CBCL/6–18 showed good internal consistency and test–retest reliability, and the factor structure has been confirmed with confirmatory factor analysis [39]. In the present study, the internal consistency of the scales was good (internalizing problems, α = 0.87, externalizing problems, α = 0.89, attention problems, α = 0.88).

### 2.4. Statistical Analysis

All measures were inspected for possible outliers, which were defined as values more than 3.29 *SD* above or below the mean [40]. Two children had extremely high scores on the internalizing scale of the CBCL, and two children on the attention problems scale. Outlying scores were changed to a value of one point above the highest non-outlying score.

The mediation–moderation model presented in Figure 1 was tested using structural equation modeling in *Mplus* version 8.3 [41]. Missing data were handled with Full Information Maximum Likelihood estimation. Maternal education was added as a covariate in all analyses because maternal education is associated with preterm birth, parenting, and behavior problems [42,43,44]. As 92% of mothers in our sample did not smoke during pregnancy, and maternal antenatal tobacco use did not correlate with birth status or child behavior problems in our study, we decided not to control for this limited amount of maternal antenatal tobacco use, in our analyses.

Parenting behavior was conceptualized as support and structure. Mother–child interaction quality was conceptualized as sensitivity and limit-setting. Internalizing, externalizing, and attention problems were added as dependent variables. Path analysis was conducted to examine the associations between birth status, parenting behavior, mother–child interaction quality, and child behavior problems. The mediating effects of support, structure, sensitivity, and limit-setting were examined using a bootstrapped mediation test; 95% bias-corrected bootstrapped confidence intervals (CIs) were used as an indicator of significance (i.e., the 95% CI does not contain zero) for all direct and indirect effects. To examine the moderating effect of birth status and whether the associations of parenting behavior and mother–child interaction quality with child behavior problems differed for MPT and FT children, a multiple group model was specified.

The model fit was evaluated with several goodness-of-fit indices: the chi-square test of model fit, with *p*-values > 0.05 indicating good fit; the Comparative Fit Index (CFI), with values >0.90 indicating satisfactory fit and values >0.95 good fit; the Tucker–Lewis index (TLI), with values >0.90 indicating good fit; the standardized root mean squared residual (SRMR), for which values of <0.08 indicate good fit; the root mean square error of approximation (RMSEA), with values of <0.06 indicating good fit [45]. Effect sizes of regression coefficients were evaluated by interpreting their standardized estimates (*β*). *β*-values of 0.10 are considered small, values of 0.30 medium, and values of ≥0.50 large [46].

## 3. Results

### 3.1. Demographic Information and Descriptive Statistics

Participants’ neonatal and demographic characteristics are presented in Table 1.

Means and standard deviations of parenting behaviors, mother–child interaction quality, and behavior problems for FT and MPT children are presented in Table 2. Correlations between study variables are shown in Table 3.

### 3.2. Associations between Birth Status, Parenting and Behavior Problems

The model presented in Figure 1 was tested to examine the associations of parenting behavior and mother–child interaction quality at 18 months with child behavior problems at 6 years. Maternal education was only significantly associated with birth status and sensitivity; associations between maternal education and all other variables were therefore removed from the model. The model showed good fit, χ^2^(6) = 8.47, *p* = 0.21, RMSEA = 0.04, CFI = 0.99, TLI = 0.92, SRMR = 0.02. Parameter estimates are reported in Table 4.

Birth status significantly predicted attention problems, but not internalizing and externalizing problems. This positive association indicates that MPT children experienced more attention problems than FT children at 6 years of age. This effect was small to moderate.

Birth status significantly predicted limit-setting at 18 months. This was a small effect, which suggests that mothers of MPT children set slightly less appropriate limits than mothers of FT children. Birth status did not significantly predict structure, support, or sensitivity at 18 months. Support, sensitivity, and limit-setting at 18 months did not significantly predict internalizing, externalizing, and attention problems at 6 years. Structure at 18 months significantly predicted internalizing and externalizing problems, but not attention problems, at 6 years. The negative associations of structure with internalizing and externalizing problems indicate that when mothers provided more structure at 18 months, children showed fewer internalizing and externalizing problems at 6 years. These effects were small to moderate.

**Figure 2:**
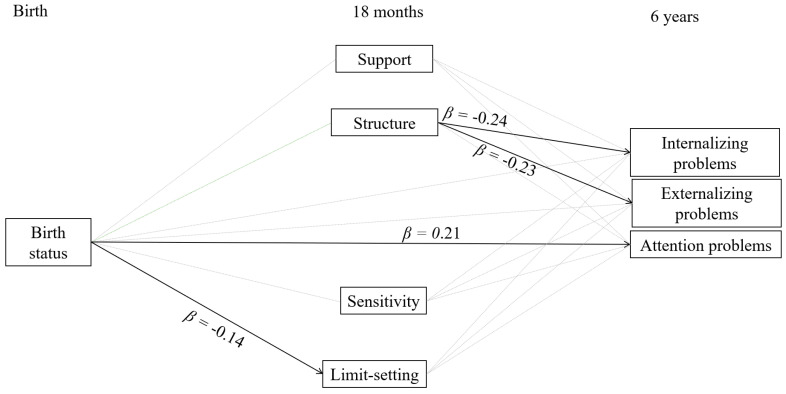
Model results. Bold lines represent significant paths. Birth status: 0 = full term, 1 = moderately preterm.

As neither structure, support, sensitivity, and limit-setting were associated with both birth status as well as behavior problems, the conditions for mediation were not met and mediation analysis was not conducted. The model results are presented in Figure 2. The model explained 9.5% of the variance in internalizing problems, 8.3% of the variance in externalizing problems, and 5.7% of the variance in attention problems.

### 3.3. Moderating Role of Birth Status

To test the hypothesis that birth status moderates the associations of parenting behavior and mother–child interaction quality with child behavior problems, birth status was removed from the model as a predictor, and was specified as the grouping variable. Separate models were specified for FT and MPT children. First, a model was specified in which all associations of support, structure, sensitivity, and limit-setting with internalizing, externalizing, and attention problems were freely estimated across both groups (M2a). Second, all associations were constrained to be equal across both groups (M2b). The chi-square difference test showed that Model 2b, in which all associations were constrained to be equal, did not fit significantly worse than the unconstrained model (M2a), Δχ^2^(19) = 8.77, *p* = 0.98. This indicates that all associations did not differ between MPT and FT children. Thus, birth status did not moderate the associations of support, structure, sensitivity, and limit-setting with internalizing, externalizing, and attention problems.

## 4. Discussion

The present study aimed to examine the longitudinal associations between birth status, parenting behavior, mother–child interaction quality, and child behavior problems in a sample of MPT and FT children. Besides premature birth, our sample of MPT children had experienced few neonatal complications. Birth status nevertheless predicted attention problems at 6 years and maternal limit-setting at 18 months. Self-reported maternal structure at 18 months predicted internalizing and externalizing problems at 6 years. Support, structure, sensitivity, and limit-setting did not mediate the association between birth status and child behavior problems. Birth status was not a significant moderator for the associations of parenting behavior and mother–child interaction quality with child behavior problems.

The MPT children in our sample experienced slightly more attention problems than FT children, which is in line with previous research [6,7,33]. Birth status did not predict internalizing and externalizing problems at 6 years. Previous research often found more substantial differences in behavior problems between MPT and FT children, particularly with regard to internalizing problems [5,6,7]. One reason for these inconsistent results might be that the present study included a sample of relatively low-risk MPT children. Specifically, this study excluded children who needed treatment at an NICU, and children with a birth weight below the 10th percentile. Most studies [5,6] did not use these exclusion criteria and studied a higher risk sample. High-risk MPT children are expected to have more behavior problems than low-risk MPT children. Future research may investigate the behavior problems of low- and high-risk MPT children by directly comparing a group of MPT children who were admitted to an NICU to a group of MPT children who did not require treatment at an NICU.

With regard to the associations of birth status with parenting behaviors and mother-child interaction quality at 18 months of corrected age, we found group differences in mother’s limit-setting. Although mothers of MPT and FT children exhibited relatively high scores in limit-setting, we found that mothers of MPT children set slightly less appropriate limits during a 15-min mother–child interaction task than mothers of FT children. This finding is consistent with previous research, showing that MPT children receive less optimal parenting than FT children [47]. However, no differences were found between MPT and FT children in other examined parenting behaviors (i.e., sensitivity, support, and structure). Thus, our hypothesis was only partly confirmed. Some studies suggest that mothers of preterm infants may start to behave more sensitively and less controlling after the first 6 months of life [47]. The timing of our study at 18 months corrected age may then explain why we did not find conclusive evidence that mothers of MPT children provide less optimal parenting than mothers of FT children.

Regarding the longitudinal associations between parenting behaviors at toddler age and child behavior problems at school age, we found that children whose mothers reported to provide more structure at 18 months of age experienced fewer internalizing and externalizing problems at 6 years of age. This result is in line with previous research showing that parenting behavior during toddlerhood predicts child behavior problems at school age [20,21]. Our finding, however, does not necessarily indicate that providing more structure directly impacts child behavior problems. Parent–child relationships are bidirectional in nature. Less optimal parenting may affect children’s behavior problems, but children’s behavior problems may also elicit less optimal parenting [48]. When (indications of) internalizing and externalizing problems are already present at 18 months of age, mothers may respond to these behavior problems by providing less structure, which could in turn exacerbate behavior problems. Future research should examine bidirectional relationships by repeatedly assessing both parenting behavior and child behavior problems over time to better understand the mechanisms involved in the development of child behavior problems.

While maternal structure was associated with internalizing and externalizing problems, we did not find an association between parenting and attention problems. Future research is needed to examine which other factors are important predictors of attention problems in MPT children. Similarly, sensitivity, limit-setting, and support at 18 months did not predict internalizing, externalizing, and attention problems at 6 years of age. Furthermore, parenting behavior and mother–child interaction quality did not mediate the association between birth status and child behavior problems. It is important to note that mothers scored generally high on the parenting constructs used in the present study, which may be explained by the relatively high level of maternal education in this sample, as high maternal education is associated with more optimal parenting behavior [42]. In samples with low variability in scores, associations between variables tend to be weaker [49]. This may explain why we did not find strong associations for most parenting constructs with birth status and behavior problems. It is also important to recognize that various methods were used to measure parenting. Structure and support were self-reported by mothers, whereas sensitivity and limit-setting were observed in a mother–child interaction task. Self-report measures cover a wider range of behaviors, time, and contexts than observations. Observations are context-specific and are restricted to one timepoint. It is unclear to what extent observations of parenting behavior generalize to other contexts [50]. In the present study, mother-reported structure was associated with later child behavior problems, whereas observed sensitivity and limit-setting were not. Future research may examine whether global self-report measures of parenting are better predictors of child behavior problems than context-specific observations.

We also expected that the behavior problems of MPT children would be differentially affected by parenting behavior and mother–child interaction quality than the behavior problems of FT children. Given that MPT children are at-risk, they may be more strongly affected by their early caregiving environment. However, in the present study all associations between the parenting variables and child behavior problems were similar for MPT and FT children. This is in contrast with previous research showing that children born very preterm and with low birth weight were more strongly affected by parenting compared with full term born and normal birth weight children [28,29]. Our sample of MPT children was relatively healthy and at low risk of neonatal complications, possibly resulting in small differences in biological risk compared with FT children. This may explain why parenting did not affect MPT children more strongly than FT children in the current study, and why we did not find evidence for the susceptibility hypothesis.

The results of this study should be interpreted in light of some limitations. First, mothers in the present sample were highly educated, almost all children were of Dutch origin, and MPT children were at relatively low biological risk. Therefore, the results may not generalize to children with less educated mothers, from different ethnic backgrounds, and MPT children at higher biological risk. Second, mothers and fathers are differentially involved in parenting, possibly impacting child behavior problems differently [51]. However, the present study included only mothers. Future studies may observe children’s interactions with both mothers and fathers, or may include multiple informants such as fathers and teachers to obtain a more complete evaluation of children’s development. Lastly, it should be noted that no strict causal conclusions can be drawn based on the correlational design of this study. Assessing parenting and behavior problems at different timepoints allowed us to infer the direction of this association. Nonetheless, there could be confounding variables that were not measured in this study, and only experimental designs can establish causality. However, it would be difficult to include all possible confounding variables in a single study. In addition, manipulating parents to use less optimal parenting behavior in an experimental setting might raise ethical concerns. Future research may examine bidirectional associations to indicate whether parenting behavior predicts child behavior, whether child behavior predicts parenting behavior, or both. A strength of the present study is its prospective longitudinal design, as long-term follow-up studies examining the development of MPT children are rare. Another strength is the multi-method design, using both questionnaires and observations. Lastly, a strength of the present study is the specific focus on MPT children, rather than combining extremely, very, and moderately preterm children into a single group.

Several implications can be made from the present study. We found elevated levels of attention problems in MPT children compared with FT children at 6 years of age. This was only a small effect, and most children in our study did not show behavior problems that were in the clinical range. However, these problems may become more prominent when children grow older, and could interfere with academic functioning [10], as MPT children are more likely to attend special education or repeat a grade [7]. This underlines the importance of early screening and, when needed, intervention for attention problems. Additionally, we found that self-reported structure in parenting by mothers at 18 months predicted fewer internalizing and externalizing problems in children at 6 years of age. Thus, structure seems to be especially important for optimal behavioral functioning of children compared with the other parenting constructs in this study. Interventions aiming to reduce internalizing and externalizing problems may educate mothers to provide more structure in parenting, which in turn may help to decrease children’s behavior problems. The fact that we could still demonstrate an association between maternal structure at 18 months and child behavior problems four and a half years later emphasizes the importance of early intervention. As associations between parenting and child behavior problems were equal for MPT and FT children, this study suggests that universal education strategies targeting parenting behavior can be recommended for both MPT as well as FT children.

## 5. Conclusions

The present study indicated longitudinal associations between birth status and maternal limit-setting during mother–child interaction at 18 months, as well as associations between maternal structure in parenting at 18 months with internalizing and externalizing behavior problems at 6 years. MPT children showed more attention problems than FT children at 6 years, but these attention problems were *not* related to mother–child interaction quality or parenting behavior at 18 months. The findings highlight the impact that perinatal risk factors, such as moderate prematurity, as well as parenting behaviors in toddlerhood, have on the development of behavior problems at early school age.

## Figures and Tables

**Table 1:** Neonatal and demographic characteristics of the full term (FT) and moderately preterm (MPT) groups.

Participants’ Characteristics	FT (*n* = 100)	MPT (*n* = 120)
Corrected age in months at wave 1		
Mean (*SD*)	17.5 (0.5)	17.5 (0.5)
Range	17–18	17–19
Corrected age in months at wave 2		
Mean (*SD*)	72.9 (0.8)	72.7 (0.6) *
Range	71.6–75.5	71.4–74.6
Gestational age		
Mean (*SD*)	39.5 (1.0)	34.7 (1.3) ***
32 weeks (%)		10.0%
33 weeks (%)		10.8%
34 weeks (%)		16.7%
35 weeks (%)		25.0%
36 weeks (%)		37.5%
37 weeks (%)	4.0%	
38 weeks (%)	10.0%	
39 weeks (%)	32.0%	
40 weeks (%)	41.0%	
41 weeks (%)	13.0%	
Birth weight in grams		
Mean (*SD*)	3578.0 (456.1)	2594.3 (515.5) ***
Range	2795–5330	1420–3850
Need for oxygen ^a^ (%)	0%	21.7% ***
Phototherapy (%)	0%	35.7% ***
Hypoglycemia (%)	0%	4.8% *
Maternal antenatal tobacco use		
No smoking during pregnancy	92.1%	91.8%
Quit smoking during pregnancy	5.0%	0.8%
Occasional smoking during pregnancy	1.0%	4.9%
Days in hospital		
Mean (*SD*)	0.41 (1.0)	11.8 (9.9) ***
Range	0–6	1–42
Gender (% boys)	45.0%	57.5%
Ethnic origin (% Dutch)	96.0%	96.7%
First born (%)	52.0%	63.3%
Maternal age at birth		
Mean (*SD*)	32.5 (4.2)	31.1 (4.5) *
Range	20–43	21–41
Maternal educational level		
Low ^b^ (%)	3.0%	9.2%
Average ^c^ (%)	11.%	35.0% ***
High ^d^ (%)	86.0%	55.8% ***

^a^ Additional oxygen right after birth, nasal cannula, and/or continuous positive airway pressure (CPAP). ^b^ No education, elementary school, special education, or lower general secondary education. ^c^ Secondary education or vocational education. ^d^ College, university or higher. * *p* < 0.05; *** *p <* 0.001.

**Table 2:** Means, standard deviations, and range for all variables.

	FT	MPT	FT		MPT	
	Mean (*SD*)	Mean (*SD*)	Min	Max	Min	Max
**Parenting behavior at 18 months**						
Support	5.08 (0.36)	5.07 (0.43)	4.23	5.85	4	6
Structure	4.95 (0.46)	4.94 (0.48)	3.83	5.92	3.67	5.83
**Mother–child interaction quality at 18 months**						
Sensitivity	4.55 (0.38)	4.50 (0.35)	3.21	5	3.21	5
Limit-setting	4.26 (0.51)	4.10 (0.60)	2.83	5	2.50	5
**Behavior problems at 6 years**						
Attention problems	52.95 (4.03)	55.12 (5.76)	50	70	50	71
Internalizing problems	45.20 (9.02)	47.60 (9.75)	33	74	33	74
Externalizing problems	45.72 (10.05)	46.12 (9.06)	34	76	34	68

**Table 3:** Correlations between study variables.

	1.	2.	3.	4.	5.	6.	7.	8.	9.
Birth status	-	−0.01	−0.01	−0.06	−0.14 *	0.13	0.02	0.21 **	−0.33 **
2. Support		-	0.28 **	−0.01	−0.10	−0.12	−0.09	−0.03	0.06
3. Structure			-	0.14 *	0.13	−0.23 **	−0.22 **	−0.10	0.10
4. Sensitivity				-	0.48 **	0.09	0.04	0	0.15 **
5. Limit-setting					-	0.04	−0.11	−0.04	0.08
6. Internalizing problems						-	0.46 **	0.37 **	−0.06
7. Externalizing problems							-	0.54 **	0.07
8. Attention problems								-	−0.15
9. Maternal education									-

* *p* < 0.05, ** *p* < 0.01. Birth status: 0 = full term, 1 = moderately preterm. Maternal education: 0 = low/average, 1 = high.

**Table 4:** Parameter estimates of the final model predicting internalizing, externalizing and attention problems.

Variable	*B*	*SE* of *B*	*β*	95% CI Low	95% CI High
Birth status → Internalizing problems	2.55	1.54	0.13	−0.51	5.67
Support → Internalizing problems	−1.60	2.05	−0.07	−5.93	1.99
**Structure → Internalizing problems**	**−4.83**	**1.77**	**−0.24**	**−8.33**	**−1.33**
Sensitivity → Internalizing problems	3.09	2.37	0.12	−1.33	7.70
Limit-setting → Internalizing problems	0.19	1.52	0.01	−2.85	3.02
Birth status → Externalizing problems	0.09	1.49	0	−2.77	2.86
Support → Externalizing problems	−1.07	1.88	−0.04	−4.86	2.87
**Structure → Externalizing problems**	**−4.63**	**1.57**	**−0.23**	**−7.49**	**−1.32**
Sensitivity → Externalizing problems	3.60	2.28	0.14	−0.86	8.05
Limit-setting → Externalizing problems	−2.73	1.55	−0.16	−5.57	0.64
**Birth status → Attention problems**	**2.14**	**0.79**	**0.21**	**0.75**	**3.92**
Support → Attention problems	−0.35	0.94	−0.03	−2.30	1.34
Structure → Attention problems	−0.98	1.01	−0.09	−3.00	1.04
Sensitivity → Attention problems	0.51	1.31	0.04	−2.13	3.01
Limit-setting → Attention problems	−0.32	0.91	−0.04	−2.14	1.39
Birth status → Support	−0.01	0.05	−0.01	−0.12	0.09
Birth status → Structure	−0.01	0.06	−0.01	−0.12	0.13
Birth status → Sensitivity	−0.02	0.05	−0.03	−0.12	0.09
Maternal education → Sensitivity	0.10	0.06	0.13	−0.01	0.21
**Birth status → Limit-setting**	**−0.16**	**0.08**	**−0.14**	**−0.30**	**−0.01**
**Support** 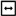 **Structure**	**0.05**	**0.01**	**0.28**	**0.03**	**0.07**
Support 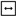 Limit-setting	−0.02	0.01	−0.10	−0.05	0.01
Support 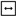 Sensitivity	0	0	−0.03	−0.02	0.01
**Sensitivity** 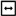 **Limit-setting**	**0.10**	**0.01**	**0.47**	**0.07**	**0.12**
Sensitivity 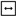 Structure	0.02	0.01	0.13	0	0.04
Limit-setting 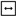 Structure	0.03	0.02	0.12	0	0.06
**Birth status** 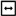 **Maternal education**	**−0.08**	**0.01**	**−0.33**	**−0.11**	**−0.05**
**Externalizing problems** 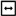 **Internalizing problems**	**35.57**	**8.02**	**0.43**	**21.74**	**54.14**
**Attention problems** 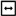 **Internalizing problems**	**15.11**	**4.73**	**0.34**	**7.47**	**26.80**
**Attention problems** 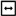 **Externalizing problems**	**24.48**	**5.46**	**0.54**	**15.50**	**38.75**

Birth status: 0 = full term, 1 = moderately preterm, maternal education: 0 = low/average, 1 = high. CI—confidence interval. 95% bias-corrected bootstrapped CIs indicate significance of the coefficients when CIs contain no zero. Bold lines represent significant associations.

## References

[1] Chawanpaiboon S., Vogel J.P., Moller A.B., Lumbiganon P., Petzold M., Hogan D., Landoulsi S., Jampathong N., Kongwattanakul K., Laopaiboon M. (2019). Global, regional, and national estimates of levels of preterm birth in 2014: A systematic review and modelling analysis. Lancet Glob. Health.

[2] Goldenberg R.L., Culhane J.F., Iams J.D., Romero R. (2008). Epidemiology and causes of preterm birth. Lancet.

[3] Shapiro-Mendoza C.K., Tomashek K.M., Kotelchuck M., Barfield W., Nannini A., Weiss J., Declercq E. (2008). Effect of late-preterm birth and maternal medical conditions on newborn morbidity risk. Pediatrics.

[4] De Jong M., Verhoeven M., van Baar A.L. (2012). School outcome, cognitive functioning, and behaviour problems in moderate and late preterm children and adults: A review. Semin. Fetal Neonatal Med..

[5] Potijk M.R., de Winter A.F., Bos A.F., Kerstjens J.M., Reijneveld S.A. (2012). Higher rates of behavioural and emotional problems at preschool age in children born moderately preterm. Arch. Dis. Child..

[6] Talge N.M., Holzman C., Wang J., Lucia V., Gardiner J., Breslau N. (2010). Late-preterm birth and its association with cognitive and socioemotional outcomes at 6 years of age. Pediatrics.

[7] van Baar A.L., Vermaas J., Knots E., de Kleine M.J., Soons P. (2009). Functioning at school age of moderately preterm children born at 32 to 36 weeks’ gestational age. Pediatrics.

[8] Gurka M.J., LoCasale-Crouch J., Blackman J.A. (2010). Long-term cognition, achievement, socioemotional, and behavioral development of healthy late-preterm infants. Arch. Pediatr. Adolesc. Med..

[9] Breslau J., Miller E., Breslau N., Bohnert K., Lucia V., Schweitzer J. (2009). The impact of early behavior disturbances on academic achievement in high school. Pediatrics.

[10] Bosquet M., Egeland B. (2006). The development and maintenance of anxiety symptoms from infancy through adolescence in a longitudinal sample. Dev. Psychopathol..

[11] Anderson V., Northam E., Wrennall J. (2019). Developmental Neuropsychology: A Clinical Approach.

[12] Walsh J.M., Doyle L.W., Anderson P.J., Lee K.J., Cheong J.L. (2014). Moderate and late preterm birth: Effect on brain size and maturation at term-equivalent age. Radiology.

[13] Rogers C.E., Anderson P.J., Thompson D.K., Kidokoro H., Wallendorf M., Treyvaud K., Roberts G., Doyle L.W., Neil J.J., Inder T.E. (2012). Regional cerebral development at term relates to school-age social–emotional development in very preterm children. J. Am. Acad. Child. Adolesc. Psychiatry.

[14] Johnson S., Evans T.A., Draper E.S., Field D.J., Manktelow B.N., Marlow N., Matthews R., Petrou S., Seaton S.E., Smith L.K. (2015). Neurodevelopmental outcomes following late and moderate prematurity: A population-based cohort study. Arch. Dis. Child. Fetal Neonatal Ed..

[15] Reidy N., Morgan A., Thompson D.K., Inder T.E., Doyle L.W., Anderson P.J. (2013). Impaired language abilities and white matter abnormalities in children born very preterm and/or very low birth weight. J. Pediatr..

[16] Sameroff A. (2010). A unified theory of development: A dialectic integration of nature and nurture. Child. Dev..

[17] Baumrind D., Damon W. (1988). Rearing competent children. Child. Development Today and Tomorrow.

[18] Suchman N.E., Rounsaville B., DeCoste C., Luthar S. (2007). Parental control, parental warmth, and psychosocial adjustment in a sample of substance-abusing mothers and their school-aged and adolescent children. J. Subst. Abuse Treat..

[19] Marcone R., Affuso G., Borrone A. (2020). Parenting styles and children’s internalizing-externalizing behavior: The mediating role of behavioral regulation. Curr. Psychol..

[20] Kok R., Linting M., Bakermans-Kranenburg M.J., van IJzendoorn M.H., Jaddoe V.W., Hofman A., Verhulst F.C., Tiemeier H. (2013). Maternal sensitivity and internalizing problems: Evidence from two longitudinal studies in early childhood. Child. Psychiatry Hum. Dev..

[21] Wang F., Christ S.L., Mills-Koonce W.R., Garrett-Peters P., Cox M.J. (2013). Association between maternal sensitivity and externalizing behavior from preschool to preadolescence. J. Appl. Dev. Psychol..

[22] Laucht M., Esser G., Schmidt M. (2001). Developmental outcome of infants born with biological and psychosocial risks. J. Child. Psychiatry.

[23] Forcada-Guex M., Pierrehumbert B., Borghini A., Moessinger A., Muller-Nix C. (2006). Early dyadic patterns of mother–infant interactions and outcomes of prematurity at 18 months. Pediatrics.

[24] Muller-Nix C., Forcada-Guex M., Pierrehumbert B., Jaunin L., Borghini A., Ansermet F. (2004). Prematurity, maternal stress and mother–child interactions. Early Hum. Dev..

[25] Segal L.B., Oster H., Cohen M., Caspi B., Myers M., Brown D. (1995). Smiling and fussing in seven-month-old preterm and full-term black infants in the still-face situation. Child. Dev..

[26] Jaekel J., Wolke D., Chernova J. (2012). Mother and child behaviour in very preterm and term dyads at 6 and 8 years. Dev. Med. Child. Neurol..

[27] Monroe S.M., Simons A.D. (1991). Diathesis-stress theories in the context of life-stress research: Implications for depressive disorders. Psychol. Bull..

[28] Gueron-Sela N., Atzaba-Poria N., Meiri G., Marks K. (2015). The caregiving environment and developmental outcomes of preterm infants: Diathesis stress or differential susceptibility effects?. Child. Dev..

[29] Jaekel J., Pluess M., Belsky J., Wolke D. (2015). Effects of maternal sensitivity on low birth weight children’s academic achievement: A test of differential susceptibility versus diathesis stress. J. Child. Psychol. Psychiatry.

[30] Belsky J., Bakermans-Kranenburg M.J., van IJzendoorn M.H. (2007). For better and for worse. Differential susceptibility to environmental influences. Curr. Dir. Psychol. Sci..

[31] Maupin A.N., Fine J.G. (2014). Differential effects of parenting in preterm and full-term children on developmental outcomes. Early Hum. Dev..

[32] Treyvaud K., Doyle L.W., Lee K.J., Ure A., Inder T.E., Hunt R.W., Anderson P.J. (2016). Parenting behavior at 2 years predicts school-age performance at 7 years in very preterm children. J. Child. Psychol. Psychiatry.

[33] Bogičević L., Verhoeven M., van Baar A.L. (2019). Toddler skills predict moderate-to-late preterm born children’s cognition and behaviour at 6 years of age. PLoS ONE.

[34] Perined (2015). Perinatal care in The Netherlands. https://www.perined.nl/producten/geboortegewichtcurven.

[35] Verhoeven M., Deković M., Bodden D., van Baar A.L. (2017). Development and initial validation of the comprehensive early childhood parenting questionnaire (CECPAQ) for parents of 1–4 year-olds. Eur. J. Dev. Psychol..

[36] Feldman R. (1998). Coding Interactive Behavior Manual.

[37] Feldman R., Klein P.S. (2003). Toddlers’ self-regulated compliance to mothers, caregivers, and fathers: Implications for theories of socialization. Dev. Psychol..

[38] Feldman R., Weller A., Sirota L., Eidelman A.I. (2003). Testing a family intervention hypothesis: The contribution of mother–infant skin-to-skin contact (Kangaroo Care) to family interaction and touch. J. Fam. Psychol..

[39] Verhulst F.C., van der Ende J. (2013). Handleiding ASEBA-Vragenlijsten voor Leeftijden 6 t/m 18 jaar: CBCL6/18, YSR en TRF.

[40] Tabachnick B.G., Fidell L.S. (2012). Using Multivariate Statistics.

[41] Muthén L.K., Muthén B.O. (1998–2017). Mplus User’s Guide.

[42] Belsky J., Bell B., Bradley R.H., Stallard N., Stewart-Brown S.L. (2007). Socioeconomic risk, parenting during the preschool years and child health age 6 years. Eur. J. Public Health.

[43] Letourneau N.L., Duffett-Leger L., Levac L., Watson B., Young-Morris C. (2013). Socioeconomic status and child development: A meta-analysis. J. Emot. Behav. Disord..

[44] Morgen C.S., Bjørk C., Andersen P.K., Mortensen L.H., Nybo Andersen A.M. (2008). Socioeconomic position and the risk of preterm birth—A study within the Danish National Birth Cohort. Int. J. Epidemiol..

[45] Hu L.T., Bentler P.M. (1999). Cutoff criteria for fit indexes in covariance structure analysis: Conventional criteria versus new alternatives. Struct. Equ. Modeling.

[46] Cohen J. (1988). Statistical Power Analysis for the Behavioral Sciences.

[47] Korja R., Latva R., Lehtonen L. (2012). The effects of preterm birth on mother–infant interaction and attachment during the infant’s first two years. Acta Obstet. Gynecol. Scand..

[48] Berg-Nielsen T.S., Vikan A., Dahl A.A. (2002). Parenting related to child and parental psychopathology: A descriptive review of the literature. Clin. Child. Psychol. Psychiatry.

[49] Goodwin L.D., Leech N.L. (2006). Understanding correlation: Factors that affect the size of r. J. Exp. Educ..

[50] Gardner F. (2000). Methodological issues in the direct observation of parent–child interaction: Do observational findings reflect the natural behavior of participants?. Clin. Child. Fam. Psychol. Rev..

[51] Cabrera N.J., Volling B.L., Barr R. (2018). Fathers are parents, too! Widening the lens on parenting for children’s development. Child. Dev. Perspect..

